# Cardioprotective effect of curcumin on myocardial ischemia/reperfusion injury: a meta-analysis of preclinical animal studies

**DOI:** 10.3389/fphar.2023.1184292

**Published:** 2023-05-22

**Authors:** Yi-Fan Zeng, Qi-Hao Guo, Xin-Yu Wei, Si-Yu Chen, Sheng Deng, Ji-Jia Liu, Ni Yin, Yan Liu, Wen-Jing Zeng

**Affiliations:** ^1^ Department of Cardiovascular Surgery, The Second Xiangya Hospital, Central South University, Changsha, Hunan, China; ^2^ Department of Pharmacy, Xiangya Hospital, Central South University, Changsha, Hunan, China; ^3^ Department of Pharmacy, Shengjing Hospital of China Medical University, Shenyang, China; ^4^ National Clinical Research Center for Geriatric Disorders, Xiangya Hospital, Central South University, Changsha, China; ^5^ Department of Pharmacy, Hunan Aerospace Hospital, Hunan Normal University, Changsha, Hunan, China

**Keywords:** curcumin, myocardial ischemia/reperfusion injury, myocardial infarction, preclinical evidence, meta-analysis

## Abstract

**Objective:** This meta-analysis aimed to determine the efficacy of curcumin in preventing myocardial ischemia/reperfusion (I/R) injury in animal models.

**Methods:** Studies published from inception to January 2023 were systematically searched in databases including PubMed, Web of Science, Embase, China’s National Knowledge Infrastructure (CNKI), Wan-Fang database, and VIP database (VIP). The SYRCLE’s RoB tool was used to determine methodological quality. Sensitivity analysis and subgroup analysis were performed when there was high heterogeneity. Publication bias was assessed using a funnel plot.

**Results:** Thirty-seven studies involving 771 animals were included in this meta-analysis with methodology quality scores ranging from 4 to 7. The results indicated that curcumin treatment significantly improved myocardial infarction size standard mean difference (SMD) = −5.65; 95% confidence interval (CI): 6.94, −4.36; *p* < 0.01; I^2^ = 90%). The sensitivity analysis for infarct size showed that the results were stable and reliable. However, the funnel plot was asymmetric. The subgroup analysis included species, animal model, dose, administration, and duration. The results showed that the subgroup dose was statistically significant between subgroups. In addition, curcumin treatment improved cardiac function, myocardial injury enzymes, and oxidative stress levels in animal models of myocardial I/R injury. The funnel plot revealed that there is publication bias for creatine kinase and lactate dehydrogenase. Finally, we performed a meta-analysis of inflammatory cytokines and apoptosis index. The results showed that curcumin treatment downregulated serum inflammatory cytokine levels and myocardial apoptosis index.

**Conclusion:** This meta-analysis suggests that curcumin has excellent potential for the treatment of myocardial I/R injury in animal models. However, this conclusion needs to be further discussed and verified in large animal models and human clinical trials.

**Systematic Review Registration:** https://www.crd.york.ac.uk/prospero/, identifier CRD42022383901.

## 1 Introduction

Acute coronary syndrome (ACS) is estimated to affect more than 7 million people annually, with a significant increase in the number of young people receiving this diagnosis (Gulati et al., 2020; Bhatt et al., 2022). The pathological basis of ACS is the rupture or invasion of coronary atherosclerotic plaque and the secondary complete or incomplete occlusive thrombosis, which fails coronary blood flow to meet the demands of myocardial metabolism, leading to sharp and temporary myocardial ischemia and hypoxia. Reperfusion therapy is the most important and effective treatment strategy known for acute myocardial infarction (AMI) (O'Gara et al., 2013). Reperfusion therapy improves myocardial ischemia and hypoxia, but it also progressively aggravates the myocardial injury and leads to myocardial cell death, which is described as myocardial ischemia/reperfusion (I/R) injury (Pagliaro et al., 2011). Oxidative stress, inflammation, and intracellular Ca^2+^ overload occur in a time-dependent manner during myocardial I/R injury, ultimately leading to cardiomyocyte death (Hausenloy and Yellon, 2013). Therefore, reducing cardiomyocyte death is an important therapeutic principle for myocardial I/R injury (Heusch, 2020).

The inherent health benefits and hypotonicity of natural products have attracted growing interest worldwide, leading to a remarkable increase in the use of nutraceuticals and dietary supplements. Turmeric, a curry spice originating in India, has attracted increasing attention in recent years for its medicinal properties. Curcumin, an extract of turmeric, is a lipophilic polyphenol with antioxidant, anti-inflammatory, and anti-fibrotic properties (Wang et al., 2012; Kotha and Luthria, 2019). Curcumin was proven to be well tolerated at high oral doses (12 g/d) and was generally considered to be safe (Lao et al., 2006; Gupta et al., 2013; Prasad et al., 2014).

Recent studies have shown that curcumin protects cardiomyocytes from myocardial I/R injury through multiple and diverse mechanisms (Wang et al., 2018a; Mokhtari-Zaer et al., 2018; Wu et al., 2021a; Pawar et al., 2022). Curcumin has been shown to improve cardiac function after myocardial I/R injury by reducing extracellular matrix degradation and inhibiting collagen synthesis via the TGFβ/Smad signaling pathway (Wang et al., 2012). In addition, curcumin attenuates oxidative damage and inhibits cardiomyocyte apoptosis by activating the JAK2/STAT3 signaling pathway, thereby ameliorating myocardial I/R injury (Liu et al., 2017a). Although the molecular mechanisms of curcumin’s cardioprotective effect in myocardial I/R injury have been partially elucidated, they are still insufficient. Therefore, there is still a long way to go before curcumin be used clinically. Furthermore, it is difficult to transfer finding of animal researches into human clinical trials. The first and most important recommendation of Rainer Spanagel’s list of 10 recommendations for improving reproducibility and translation is to perform a preclinical meta-analysis (Spanagel, 2022). Therefore, this preclinical meta-analysis aimed to determine whether curcumin has a cardioprotective role in animal models of myocardial I/R injury.

## 2 Methods

This meta-analysis has been registered in PROSPERO (ID: CRD42022383901). This study was conducted in Covidence from literature selection to data extraction.

### 2.1 Search strategy

Six databases, including Web of Science, PubMed, Embase, China’s National Knowledge Infrastructure (CNKI), the Wan-Fang database, and the VIP database (VIP), were searched systematically between database inception and January 2023 without language restrictions. The keywords mainly used were “myocardial infarction”, “myocardial ischemia”, “myocardial I/R″, “myocardial I/R injury”, “myocardial ischemia-reperfusion injury”, “myocardial ischemia-reperfusion”, and “curcumin”.

### 2.2 Study selection

The included studies met the following criteria: (Bhatt et al., 2022): myocardial I/R injury experimental models: left anterior descending (LAD) ligation, injecting isoprenaline (ISO) intravenously, or endovascular embolization; (Gulati et al., 2020); Treatment: curcumin was the only intervention with a control group receiving placebo fluid or no treatment at all; and (O'Gara et al., 2013) Data: detailed data of the primary or secondary outcomes in the articles.

Exclusion Criteria were as follows: (Bhatt et al., 2022): no detailed data was provided, (Gulati et al., 2020), no animal model, (O'Gara et al., 2013), without a control group, (Pagliaro et al., 2011), Curcumin is not the only intervention, (Hausenloy and Yellon, 2013), *in vitro* studies, (Heusch, 2020), article types: review, conference abstract, case reports, meta-analysis and clinical trials.

### 2.3 Data extraction

Two independent reviewers screened relevant articles for titles and abstracts, viewed full texts, and then extracted data from identified studies by Covidnce in the same standard form. The search results were then checked by the third reviewer. Any disagreements therein were resolved with discussion and adjudication by group discussion.

Information extracted includes as follows: (Bhatt et al., 2022): Author information: the first author, publication year, and country; (Gulati et al., 2020); Animal information: species, sex, and sample size; (O'Gara et al., 2013); Animal model: anesthetic and model methods; (Pagliaro et al., 2011); Drug administration: method, dosage, and duration of administration; (Hausenloy and Yellon, 2013); Outcome record: the mean and standard deviation of the primary outcomes. Primary outcomes include myocardial infarction size (IS). Secondary outcomes include left ventricular ejection fraction (LVEF), left ventricular fractional shortening (LVFS), left ventricular developed pressure (LVDP), maximum 1st derivative of developed pressure (dP/dt max), serum creatine kinase (CK), serum creatine kinase-MB (CK-MB), lactate dehydrogenase (LDH), malondialdehyde (MDA), superoxide dismutase (SOD), catalase (CAT), interleukin-1β (IL-1β), interleukin-6 (IL-6), tumor necrosis factor-α (TNF-α), and apoptosis index. Primary and secondary outcomes were extracted in detail. When different doses of the curcumin were used, data in the highest dose group was extracted. When there are multiple time points after myocardial ischemia, only the last time point is recorded.

### 2.4 Quality assessment

The quality assessment of the included studies was independently evaluated by two authors using the SYRCLE’s RoB tool (Hooijmans et al., 2014).

### 2.5 Statistical analysis

The summary statistics were considered by standard mean difference (SMD) and 95% confidence interval (CI). The Cochran Q test and the I^2^ statistics were used for the study heterogeneity assessment. Different effect models were used according to the heterogeneity. The random effect model was used when I^2^>50%, on the contrary, the fixed effect model was used. All statistics were analyzed by R (Version 4.2.2). Subgroup analysis and sensitivity analysis were conducted to find the source of heterogeneity. Publication bias was evaluated, when more than 10 studies were included, by funnel plot. *p* < 0.05 indicated that the difference was statistically significant.

## 3 Results

### 3.1 Study selection

**FIGURE 1:**
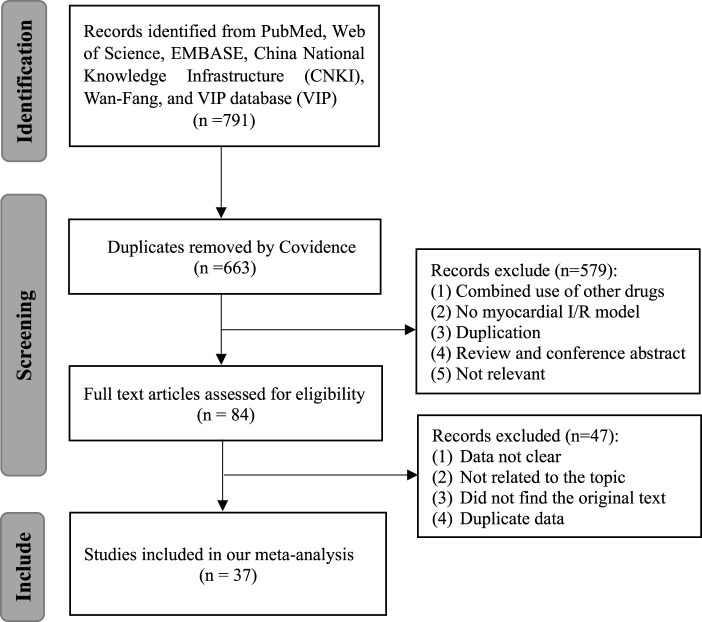
Flow diagram of database searches and study selection.

Seven hundred and ninety-one articles were involved after the primary retrieval. One hundred and twenty-eight duplicate articles were excluded by Covidence. After carefully reading the abstracts and titles, 579 articles were excluded. Forty-seven studies were excluded after full-text articles were assessed due to articles with unclear data, irrelevant to our topic, the original text being unavailable, and duplicate data. Finally, a total of 37 studies involving 755 animals were included in the present meta-analysis (Nirmala and Puvanakrishnan, 1996; Manikandan et al., 2004; Cheng et al., 2005; Ansari et al., 2007; Tanwar et al., 2010; Zhao et al., 2010; Wu et al., 2011; Duan et al., 2012; Izem-Meziane et al., 2012; Jeong et al., 2012; Kim et al., 2012; Yang et al., 2012; Wang et al., 2013; Yang et al., 2013; Su et al., 2014; Wang et al., 2014; Yao and Jiang, 2014; Xiao et al., 2016a; Geng et al., 2016; Gu et al., 2016; Sun et al., 2016; Liu et al., 2017b; Liu et al., 2017c; Wang et al., 2018b; Chen et al., 2018; Liu et al., 2018; Boarescu et al., 2019a; Li et al., 2019a; Boarescu et al., 2019b; Javanmard et al., 2019; Rahnavard et al., 2019; Li et al., 2020a; Ding et al., 2020; Jo et al., 2020; Wu et al., 2021b; Yan et al., 2021; Zhao et al., 2022). The PRISMA flow diagram is shown in Figure 1.

### 3.2 Characteristics of included studies

In terms of species, 21 studies used Sprague-Dawley rats, 10 studies used Wistar rats, and 6 studies used C57BL/6 mice. In terms of gender, only 3 studies used female animals and all the others were male. The methods used to establish animal models of myocardial I/R injury included LAD ligation in 26 studies, ISO injection in 10 studies, and coronary microembolization in 1studies.

Curcumin was administered by multiple routines including oral in 20 studies, intraperitoneal in 7 studies, intravenous in 4 studies, and perfused in 6 studies. Additionally, the duration of curcumin treatment varied from 1 min to 12 weeks. The publication year of the included studies varied between 1996 and 2022. Twenty-four of 37 studies were conducted in China, 4 studies in India, 3 studies in Korea, 2 studies each in Iran or Romania, and 1 study each in France or the United States. All the basic information about the included studies in this meta-analysis is shown in Table 1.

**TABLE 1:** Baseline characteristics of included studies.

Author	Year	Country	Species	N (CCM/Ctrl)	Anesthetic	Model method	Methods to administration (dose)	Duration	Outcomes
Ansari et al.,	2007	India	male Wistar rats	8/8	—	ISO	Oral (200 mg/kg/d)	20 days	1.CK, 2.LDH, 3.CAT.
Boarescu et al.,	2019	Romania	male Wistar rats	7/7	—	ISO	Oral (200 mg/kg/d)	2 weeks	1.CK, 2.CK-MB, 3.LDH, 4.MDA, 5.IL-1β, 6.IL-6, 7.TNF-α
Boarescu et al.,	2019	Romania	female Wistar rats	7/7	—	ISO	Oral (200 mg/kg/d)	2 weeks	1.CK, 2.CK-MB, 3.MDA, 4.IL-1β, 5.IL-6, 6.TNF-α
Chen et al.,	2018	China	male SD rats	20/20	Pentobarbital sodium	Ischemia 30 min then reflow 6 h	Intravenous (20 mg/kg)	1 min	1.LVDP
									2.+dP/dt max
									3.-dP/dt max, 4.CK, 5.LDH.
Cheng et al.,	2005	China	male SD rats	10/10	Pentobarbital sodium (0.9 mg/kg)	Ischemia 60 min then reflow 30 min	Intravenous (40 mg/kg)	5 min	1. Infarction size
Ding et al.,	2020	China	male SD rats	10/10	Phenobarbital	Ischemia 30 min then reflow 60 min	Oral (400 mg/kg/d)	12 weeks	1.CK, 2.CK-MB, 3.LDH, 4.SOD.
Duan et al.,	2012	China	male SD rats	8/8	Pentobarbital sodium (30 mg/kg)	Ischemia 60 min then reflow 60 min	Perfused (1uM)	10 min	1.Infarction size, 2.LVDP
									3.+dP/dt max
Geng et al.,	2016	China	male C57BL/6 J	8/8	5.0% isoflurane	LAD ligation	Oral (50 mg/kg)	1 week	1.Infarction size
Gu et al.,	2016	China	male C57BL/6 mice	10/10	Pentobarbital sodium (0.5 mg/kg)	Ischemia 30 min then reflow 4 h	Intraperitoneal (100 mg/kg)	30 min	1.Infarction size
									2.TNF-α
Izem-Meziane et al.,	2012	France	male Wistar rats	5/5	—	ISO	Intraperitoneal (60 mg/kg/d)	2 days	1.CAT, 2.Apoptosis Index
Javanmard et al.,	2019	Iran	male Wistar rats	6/6	—	ISO	Oral (80 mg/kg/d)	12 days	1.Infarction size, 2.CK, 3.LDH, 4.MDA, 5.SOD
									6.Apoptosis Index
Jeong et al.,	2012	Korea	male SD rats	7/7	Pentobarbital (60 mg/kg)	Ischemia 30 min then reflow 2 h	Intraperitoneal (100 mg/kg/d)	20 min	1.Infarction size
Jo et al.,	2020	Korea	male SD rats	8/8	Alfaxalone (50 mg/kg) and xylazine (5 mg/kg)	Ischemia 30 min then reflow 4 days	Oral (50 mg/kg/d)	5 days	1.Infarction size, 2.LVEF, 3.LVFS, 4.CK-MB, 5.LDH.
Kim et al.,	2012	Korea	male Wistar rats	3/4	Ketamine (50 mg/kg) and xylazine (5 mg/kg)	Ischemia 30 min then reflow 14 days	Oral (300 mg/kg/d)	2 weeks	1.Infarction size, 2.LVEF, 3.LVFS, 4.+dP/dt max
									5.-dP/dt max
Li et al.,	2019	China	male C57BL/6 J	32/32	sodium pentobarbital (50 mg/kg)	LAD ligation	Oral (100 mg/kg/d)	4 weeks	1. Infarction size, 2. LVEF
									3. LVFS.
Li et al.,	2020	China	male SD rats	10/10	Isoflurane	Ischemia 30 min then reflow 2 h	Intraperitoneal (20 mg/kg)	4 h	1.LVDP, 2.+dP/dt max, 3.-dP/dt max, 4.CK, 5.LDH, 6.MDA, 7.SOD, 8.Apoptosis Index
Liu et al.,	2017	China	male SD rats	10/10	Ketamine (90 mg/kg) and xylazine (10 mg/kg)	Ischemia 60 min then reflow 3 h	Oral (30 mg/kg/d)	20 days	1.Infarction size, 2.LVDP, 3.+dP/dt max, 4.-dP/dt max, 5.MDA.
Liu et al.,	2017	China	male SD rats	10/10	chloral hydrate (350 mg/kg)	Ischemia 30 min then reflow 30 min	Perfused (0.5 mg/kg)	30 min	1.Infarction size
Liu et al.,	2018	China	male SD rats	10/10	pentobarbital (30–40 mg/kg)	Endovascular embolization by microspheres	Oral (150 mg/kg/d)	5 days	1.LVEF, 2.LVFS, 3.IL-1β, 4.TNF-α, 5.Apoptosis Index
Manikandan et al.,	2004	India	female Wistar rats	6/6	—	ISO	Oral (15 mg/kg/d)	30 min	1.CAT.
Nirmala et al.,	1996	India	female Wistar rats	6/6	—	ISO	Oral (200 mg/kg/d)	4 days	1.CK, 2.LDH, 3.CAT.
Rahnavard et al.,	2019	Iran	male Wistar rats	6/6	—	ISO	Oral (50 mg/kg/d)	9 days	1.Infarction size, 2.CK, 3.LDH, 4.MDA, 5.SOD.
Su et al.,	2014	China	male SD rats	10/10	NA	Ischemia 30 min then reflow 2 h	Oral (200 mg/kg/d)	4 weeks	1.CK-MB.
Sun et al.,	2016	China	male SD rats	10/10	Chloral hydrate	Ischemia 45 min then reflow 2 h	Intraperitoneal (50 mg/kg)	45 min	1.+dP/dt max
									2.-dP/dt max, 3.CK, 4.LDH.
Tanwar et al.,	2010	India	male Wistar rats	10/10	—	ISO	Oral (400 mg/kg/d)	2 weeks	1.CAT
Wang et al.,	2013	China	male SD rats	8/8	Pentobarbital (30 mg/kg)	Ischemia 30 min then reflow 150 min	Perfused (1uM)	60 min	1.Infarction size, 2.LVDP, 3.+dP/dt max
Wang et al.,	2014	United States	male SD rats	8/8	ketamine (90 mg/kg) and zylaxine (10 mg/kg)	Ischemia 30 min then reflow 3 h	Oral (150 mg/kg/d)	5 days	1.Infarction size
									2.IL-6
									3.TNF-a
Wang et al.,	2018	China	male SD rats	6/6	sodium pentobarbital (50 mg/kg)	Ischemia 45 min then reflow 2 h	Perfused (1uM)	120 min	1.Infarction size
									2.LDH
									3.Apoptosis Index
Wu et al.,	2011	China	male SD rats	10/10	Sodium pentobarbital (0.9 mg/kg)	Ischemia 60 min then reflow 30 min	Oral (40 mg/kg)	30 min	1.Infarction size, 2.LDH, 3.MDA, 4.SOD.
Wu et al.,	2021	China	male SD rats	10/10	10% chloral hydrate (3.5 mL/kg)	Ischemia 30 min then reflow 3 h	Intraperitoneal (100 mg/kg/d)	30 min	1.Infarction size, 2.CK-MB, 3.LDH, 4.MDA, 5.SOD.
Xiao et al.,	2016	China	male C57BL/6 J mice	12/12	2% isoflurane	LAD ligation	Oral (100 mg/kg)	5 weeks	1.Infarction size
Yan et al.,	2021	China	male C57BL/6 J mice	33/33	ketamine (50 mg/kg) and pentobarbital sodium (50 mg/kg)	LAD ligation	Intraperitoneal (100 mg/kg/d)	6 weeks	1.Infarction size, 2.LVEF, 3.LVFS, 4.IL-1β, 5.IL-6, 6.TNF-α
Yang et al.,	2012	China	male SD rats	8/8	Pentobarbital (30 mg/kg)	Ischemia 45 min then reflow 60 min	Perfused (1uM)	5 min	1.Infarction size, 2.LVDP
									3.+dP/dt max
Yang et al.,	2013	China	male SD rats	8/8	sodium pentobarbital (50 mg/kg)	Ischemia 45 min then reflow 60 min	Perfused (1uM)	60 min	1.Infarction size, 2.Apoptosis Index
Yao et al.,	2014	China	male SD rats	12/12	Sodium pentobarbital (0.9 mg/kg)	Ischemia 30 min then reflow 6 h	Intravenous (20 mg/kg)	1 min	1.LVDP
									2.+dP/dt max
									3.-dP/dt max, 4.CK, 5.LDH, 6.MDA, 7.SOD.
Zhao et al.,	2010	China	male SD rats	20/20	Urethane (0.1 mL/kg)	Ischemia 30 min then reflow 2 h	Intravenous (20 mg/kg)	30 min	1.CK, 2.LDH.
Zhao et al.,	2022	China	male C57BL/6 J mice	5/5	1.5%–2% isoflurane	LAD ligation	Oral (100 mg/kg/d)	4 weeks	1.Infarction size, 2.LVEF, 3.LVFS.

Note: SD rats, Sprague-Dawley rats; ISO, isoproterenol; LAD, left anterior descending branch; LVEF, left ventricular ejection fraction; LVFS, left ventricular fractional shortening; LVDP, left ventricular diastolic pressure; dP/dT max, maximum 1st derivative of developed pressure; CK, creatine kinase; CK-MB, creatine kinase isoenzyme; LDH, lactic dehydrogenase; MDA, malondialdehyde; SOD, superoxide dismutase; CAT, catalase; IL, interleukin; TNF, tumor necrosis factor.

The studies included in this meta-analysis scored between 4 and 7 on the quality assessment. The details of the methodological quality of the included literature are shown in Table 2. The brief molecular mechanism by which curcumin protects cardiomyocytes from myocardial I/R injury is summarized in Table 3.

**TABLE 2:** Quality assessment of included studies by the SYRCLE’s RoB tool.

Study	Published year	A	B	C	D	E	F	G	H	I	J	Score
Ansari et al.,	2007		√	√			√		√	√		5
Boarescu et al.,	2019		√	√			√		√	√	√	6
Boarescu et al.,	2019		√	√			√		√	√		5
Chen et al.,	2018		√	√			√		√	√	√	6
Cheng et al.,	2005		√	√			√		√	√	√	6
Ding et al.,	2020		√	√			√		√	√	√	6
Duan et al.,	2012		√	√			√		√	√		5
Geng et al.,	2016		√	√			√		√	√	√	6
Gu et al.,	2016		√	√			√		√	√	√	6
Izem-Meziane et al.,	2012		√	√			√		√	√	√	6
Javanmard et al.,	2019		√	√			√		√	√	√	6
Jeong et al.,	2012		√	√			√		√	√		5
Jo et al.,	2020		√	√			√		√	√	√	6
Kim et al.,	2012		√	√			√		√			4
Li et al.,	2020		√	√			√		√	√	√	6
Li et al.,	2019		√	√			√			√	√	5
Liu et al.,	2017		√	√			√		√	√	√	6
Liu et al.,	2017		√	√			√		√		√	5
Liu et al.,	2018		√	√			√		√	√	√	6
Manikandan et al.,	2004		√	√			√		√	√		5
Nirmala et al.,	1996		√	√			√		√	√	√	6
Rahnavard et al.,	2019		√	√			√		√	√	√	6
Su et al.,	2014		√	√			√		√	√	√	6
Sun et al.,	2016		√	√			√			√		4
Tanwar et al.,	2010		√	√			√		√	√	√	6
Wang et al.,	2013		√	√			√		√	√	√	6
Wang et al.,	2014		√	√			√		√	√		5
Wang et al.,	2018		√	√			√		√	√	√	6
Wu et al.,	2011		√	√			√		√	√		5
Wu et al.,	2021		√	√			√		√	√	√	6
Xiao et al.,	2016		√	√			√		√	√	√	6
Yan et al.,	2021		√	√			√		√	√	√	6
Yang et al.,	2012		√	√			√		√	√	√	6
Yang et al.,	2013		√	√			√		√	√		5
Yao et al.,	2014		√	√			√		√	√	√	6
Zhao et al.,	2010		√	√			√		√	√		5
Zhao et al.,	2022		√	√		√	√		√	√	√	7

Note: A, sequence generation; B, baseline characteristics; C, allocation concealment; D, random housing; E, blinding investigators; F, random outcome assessment; G, blinding outcome assessor; H, incomplete outcome data; I, selective outcome reporting; J, other sources of bias.

**TABLE 3:** The molecular and cellular mechanisms underlying the cardioprotective effect of Curcumin treatment in myocardial ischemia/reperfusion injury.

Author	Published year	Proposed mechanisms
Ansari et al.,	2007	Enhance the antioxidant capability
Boarescu et al.,	2019	Enhance antioxidative and anti-inflammatory effects
Boarescu et al.,	2019	Limit the increase in inflammatory cytokine levels and MMP2/9 expression
Chen et al.,	2018	Increase HO-1 activity and expression
Cheng et al.,	2005	Reduce lipid peroxide and increase production of endogenous antioxidative substances
Ding et al.,	2020	Reduce myocardial enzyme release and enhance antioxidant capacity
Duan et al.,	2012	Activate of the JAK2/STAT3 signaling pathway
Geng et al.,	2016	Upregulate miR-7a/b expression and downregulate of SP1 expression
Gu et al.,	2016	Inhibit TLR4/NF-kappaB signaling pathway
Izem-Meziane et al.,	2012	Prevent mitochondrial damaging and mitochondrial permeability transition pore (mPTP) opening
Javanmard et al.,	2019	NA
Jeong et al.,	2012	Activate RISK/GSK-3β and inhibit p38 and JNK.
Jo et al.,	2020	NA
Kim et al.,	2012	Selective inhibit of TLR2
Li et al.,	2019	Immunomodulatory effect
Li et al.,	2020	NA
Liu et al.,	2017	Stimulate JAK2/STAT3 signal pathway, decrease oxidative damage, and inhibit myocardium apoptosis
Manikandan et al.,	2004	Inhibit xanthine dehydrogenase/xanthine oxidase (XD/XO) conversion and resultant superoxide anion production
Nirmala et al.,	1996	Increase activity of cathepsin D in mitochondrial, lysosomal, and microsomal fractions
Rahnavard et al.,	2019	Reduce oxidative status by reducing SOD and MDA contents
Su et al.,	2014	NA
Sun et al.,	2016	Suppresses ROS-mediating mitochondrial apoptosis
Tanwar et al.,	2010	Stabilize cytoskeleton structure and increase Hsp27 expression
Wang et al.,	2013	Activate Notch signaling pathway
Wang et al.,	2014	Downregulate expression of EGR-1 and inhibit inflammation-mediated processes
Wang et al.,	2018	Activate SIRT3
Wu et al.,	2011	NA
Wu et al.,	2021	Downregulate the expression of Bax and upregulate the expression of Bcl2, p-mTOR and p-AKT
Yan et al.,	2021	Suppress inflammation by modulating macrophage polarization via the AMPK pathway
Yang et al.,	2012	Activate anti-apoptotic signaling pathway
Yang et al.,	2013	Activate of SIRT1 signaling and the alleviate of mitochondrial oxidative damage
Yao et al.,	2014	Anti-oxidative injury and upregulate activity and expression of HO-1 protein
Zhao et al.,	2010	NA
Zhao et al.,	2022	Inhibit of macrophage-fibroblast crosstalk and activate of IL18-TGFβ1-p-SMAD2/3 signaling pathway in cardiac fibroblasts

### 3.3 Outcome measures

#### 3.3.1 Myocardial infarct size

**FIGURE 2:**
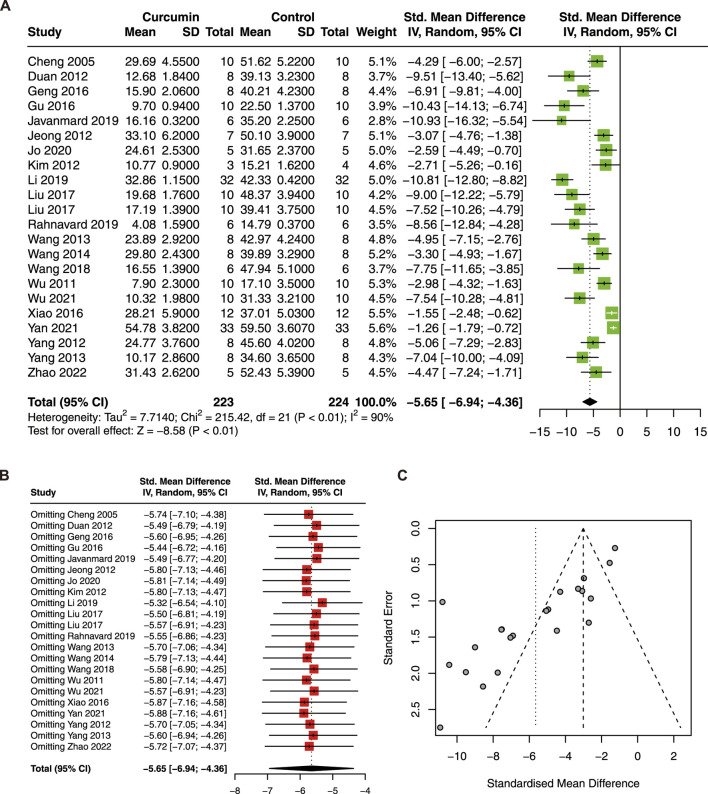
**(A)** Forest plot showing cardioprotective effects of curcumin on myocardial infarction size (IS) in myocardial ischemia/reperfusion injury animal model; **(B)** sensitive analysis of IS; **(C)** funnel plot of IS.

Twenty-two studies with 447 animals were included in this meta-analysis to evaluate the effect of curcumin on IS (Cheng et al., 2005; Duan et al., 2012; Jeong et al., 2012; Kim et al., 2012; Wang et al., 2013; Geng et al., 2016; Gu et al., 2016; Liu et al., 2017b; Liu et al., 2017c; Li et al., 2019a; Javanmard et al., 2019; Rahnavard et al., 2019; Jo et al., 2020). The result showed that curcumin treatment significantly reduced IS (SMD = −5.65; 95% CI: −6.94, −4.36; *p* < 0.01; I^2^ = 90%) in the animal model of myocardial I/R injury (Figure 2A).

Due to high heterogeneity, sensitivity analysis, funnel plot, and subgroup analysis were conducted. The result of the sensitivity analysis revealed that omitting a single study did not significantly change the pooled estimate of IS (Figure 2B). However, the funnel plot of IS was asymmetric (Figure 2C). To explore the source of heterogeneity, species, animal models, dose, administration, and duration were included in the subgroup analysis. The results showed that the subgroup dose was statistically significant between subgroups (Table 4). Furthermore, significant decreases in heterogeneity were found in the ISO model and in the administration of perfusion.

**TABLE 4:** Subgroup analysis of pooled estimates of infarct size.

Subgroup	No. of studies	SMD	95% CI	P value between subgroups	heterogeneity within subgroups	
					I^2^(%)	P value
**Species**				0.81		
SD rats	13	-5.34	-6.60, -4.08		72	< 0.01
Wistar rats	3	-6.98	-11.96, -1.99		81	< 0.01
C57BL/6 mice	6	-5.73	-9.14, -2.32		96	< 0.01
**Model**				0.08		
I/R	15	-5.48	-6.77, -4.19		74	< 0.01
MI	5	-4.90	-8.46, -1.33		96	< 0.01
ISO	2	-9.48	-12.83; -6.13		0	0.50
**Dose**				<0.01		
Perfused	6	-6.50	-7.86, -5.14		26	0.24
≤50mg/kg	6	-5.32	-7.49, -3.14		77	<0.01
50-100mg/kg	8	-5.94	-8.84, -3.04		95	<0.01
>100mg/kg	2	-3.13	-4.50, -1.75		90	<0.01
**Drug Delivery**				0.26		
Perfused	6	-6.50	-7.86, -5.14		26	0.24
Intraperitoneal	4	-5.30	-9.33, -1.28		93	< 0.01
Oral	11	-5.46	-7.51, -3.42		90	< 0.01
Intravenous	1	-4.29	-6.00, -2.57		/	/
**Duration**				0.37		
<1day	11	-5.96	-7.40, -4.52		74	< 0.01
1day-1week	3	-4.02	-6.35, -1.69		68	0.05
>1week	8	-5.84	-8.73, -2.95		94	< 0.01

Note: SMD, standardized mean difference; SD rats, Sprague-Dawley rats; I/R, ischemia/reperfusion; MI, myocardial infarction; ISO, isoproterenol.

#### 3.3.2 Cardiac function

To determine the association between curcumin treatment and cardiac function in the animal model of myocardial I/R injury, LVEF, LVFS, LVDP, +dP/dt max, and -dP/dt max were analyzed. Six studies with 183 animals for LVEF and LVFS (Kim et al., 2012; Liu et al., 2018; Li et al., 2019a; Jo et al., 2020; Yan et al., 2021; Zhao et al., 2022), 7 studies with 152 animals for LVDP (Duan et al., 2012; Yang et al., 2012; Wang et al., 2013; Yao and Jiang, 2014; Liu et al., 2017b; Chen et al., 2018; Li et al., 2020a), 9 studies with 187 animals for + dP/dt max (Duan et al., 2012; Kim et al., 2012; Yang et al., 2012; Wang et al., 2013; Yao and Jiang, 2014; Sun et al., 2016; Liu et al., 2017b; Chen et al., 2018; Li et al., 2020a), and 6 studies with 139 animals for -dP/dt max (Kim et al., 2012; Yao and Jiang, 2014; Sun et al., 2016; Liu et al., 2017b; Chen et al., 2018; Li et al., 2020a) were included in this meta-analysis.

**FIGURE 3:**
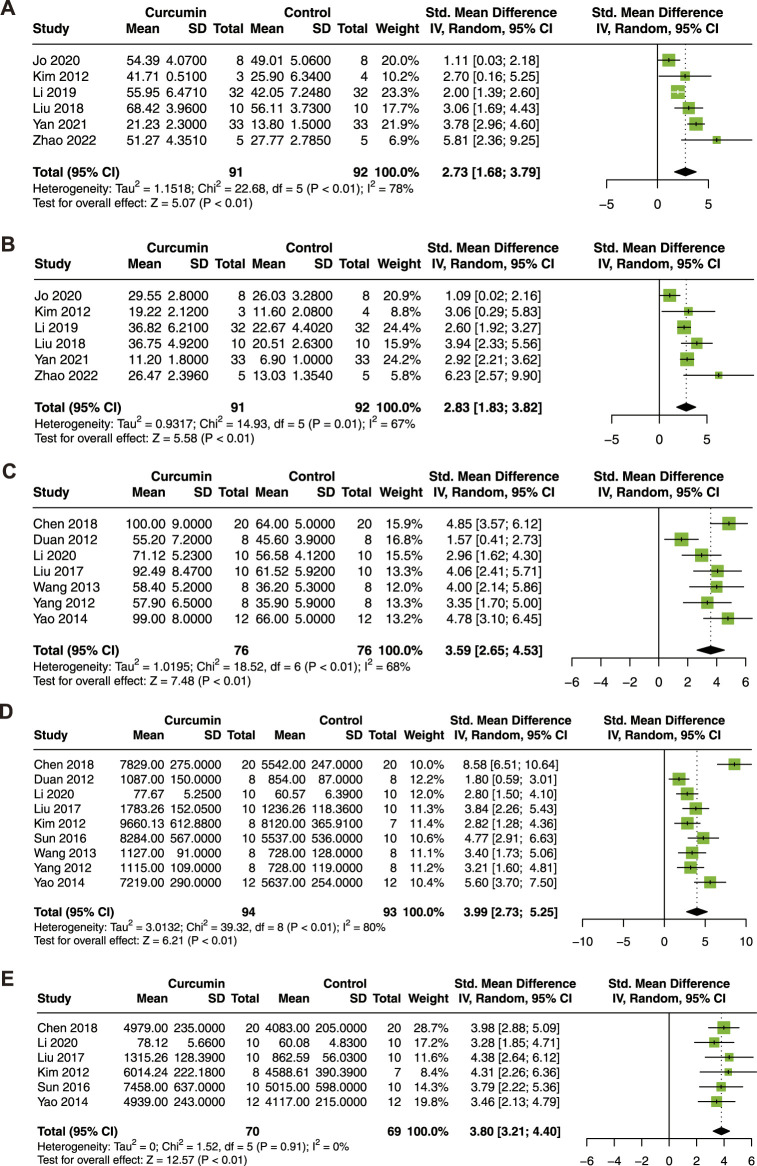
Forest plot showing cardioprotective effects of curcumin on LVEF **(A)**, LVFS **(B)**, LVDP **(C)**, +dP/dt max **(D)**, and -dP/dt max **(E)** in myocardial ischemia/reperfusion injury animal model. LVEF, left ventricular ejection fraction; LVFS, left ventricular fractional shortening; LVDP, left ventricular diastolic pressure; dP/dt max, maximum 1st derivative of developed pressure.

As shown in Figure 3, curcumin treatment exhibited a significant improvement in LVEF (SMD = 2.73; 95% CI: 1.68, 3.79; *p* < 0.01; I^2^ = 78%; Figure 3A), LVFS (SMD = 2.83; 95% CI: 1.83, 3.82; *p* = 0.01; I^2^ = 67%; Figure 3B), LVDP (SMD = 3.59; 95% CI: 2.65, 4.53; *p* < 0.01; I^2^ = 68%; Figure 3C), +dP/dt max (SMD = 3.99; 95% CI: 2.73, 5.25; *p* < 0.01; I^2^ = 80%; Figure 3D), and -dP/dt max (SMD = 3.80; 95% CI: 3.21, 4.40; *p* < 0.01; I^2^ = 0%; Figure 3E). In conclusion, curcumin treatment significantly improved cardiac function in animal models of myocardial I/R injury.

#### 3.3.3 Myocardial enzyme

Then, serum biomarkers of myocardial injury, including CK, CK-MB, and LDH, were analyzed. Twelve studies with 244 animals for CK (Nirmala and Puvanakrishnan, 1996; Ansari et al., 2007; Zhao et al., 2010; Yao and Jiang, 2014; Sun et al., 2016; Chen et al., 2018; Boarescu et al., 2019a; Boarescu et al., 2019b; Javanmard et al., 2019; Rahnavard et al., 2019; Li et al., 2020a; Ding et al., 2020), 6 studies with 94 animals for CK-MB (Su et al., 2014; Boarescu et al., 2019a; Boarescu et al., 2019b; Ding et al., 2020; Jo et al., 2020; Wu et al., 2021b), and 16 studies with 304 animals for LDH (Nirmala and Puvanakrishnan, 1996; Ansari et al., 2007; Zhao et al., 2010; Wu et al., 2011; Wang et al., 2013; Yao and Jiang, 2014; Sun et al., 2016; Wang et al., 2018b; Chen et al., 2018; Boarescu et al., 2019a; Javanmard et al., 2019; Rahnavard et al., 2019; Li et al., 2020a; Ding et al., 2020; Jo et al., 2020; Wu et al., 2021b) were included in this meta-analysis.

**FIGURE 4:**
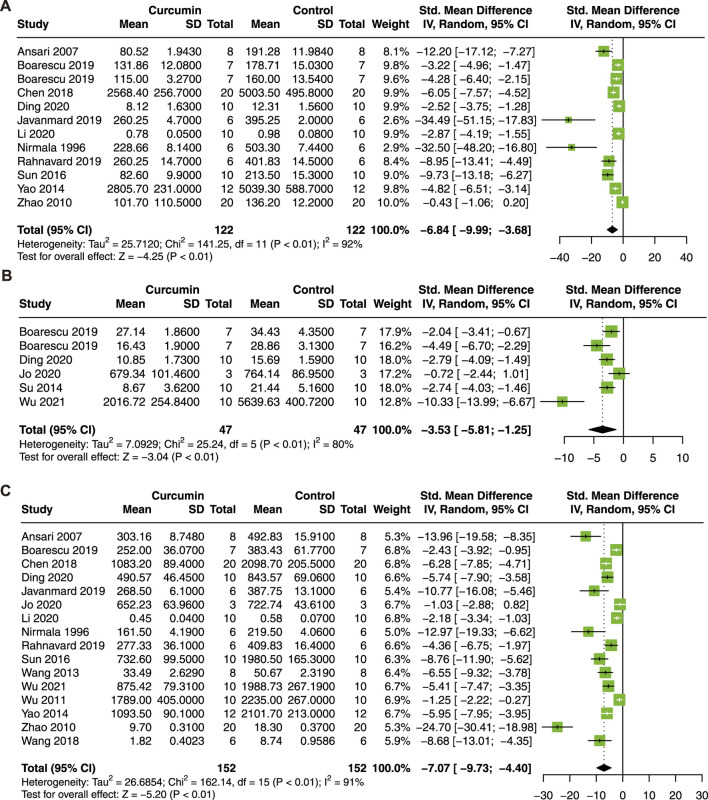
Forest plot showing cardioprotective effects of curcumin on CK **(A)**, CK-MB **(B)**, and LDH **(C)** in myocardial ischemia/reperfusion injury animal model. CK, creatine kinase; CK-MB, creatine kinase isoenzyme; LDH, lactic dehydrogenase.

As a result, curcumin treatment presented a significant effect on the reduction of serum CK (SMD = −6.84; 95% CI: −9.99, −3.68; *p* < 0.01; I^2^ = 92%; Figure 4A), CK-MB (SMD = −3.53; 95% CI: −5.81, −1.25; *p* < 0.01; I^2^ = 80%; Figure 4B), and LDH (SMD = −7.07; 95% CI: −9.73, −4.40; *p* < 0.01; I^2^ = 91%; Figure 4C) in animals with myocardial I/R injury.

**FIGURE 5:**
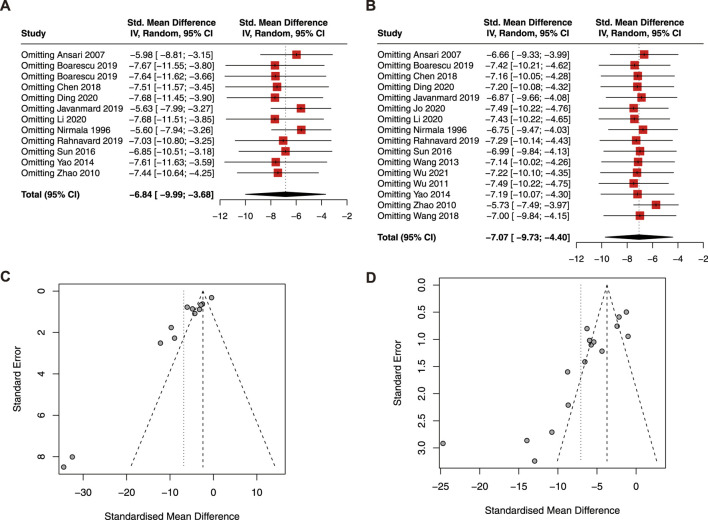
Sensitive analysis of CK **(A)** and LDH **(B)**; funnel plot of CK **(C)** and LDH **(D)**. CK, creatine kinase; LDH, lactic dehydrogenase.

The result of the sensitivity analysis revealed that the pooled estimate of CK (Figure 5A) and LDH (Figure 5B) was stable and reliable. However, the funnel plots of CK (Figure 5C) and LDH (Figure 5D) were asymmetric, indicating publication bias.

#### 3.3.4 Oxidative stress levels

MDA is a crucial indicator of tissue oxidative stress and is a degradation product of lipid peroxidation (Idris et al., 2001). CAT level is known to decrease after myocardial I/R injury (Jin et al., 2017). In addition, SOD is an important antioxidant enzyme. To investigate the association between curcumin treatment and oxidative stress levels, MDA, SOD, and CAT were analyzed. Nine studies with 156 animals for serum MDA (Wu et al., 2011; Yao and Jiang, 2014; Liu et al., 2017b; Boarescu et al., 2019a; Boarescu et al., 2019b; Javanmard et al., 2019; Rahnavard et al., 2019; Li et al., 2020a; Wu et al., 2021b), 5 studies with 104 animals for serum SOD (Wu et al., 2011; Yao and Jiang, 2014; Li et al., 2020a; Ding et al., 2020; Wu et al., 2021b), and 5 studies with 70 animals for CAT in heart tissue (Nirmala and Puvanakrishnan, 1996; Manikandan et al., 2004; Ansari et al., 2007; Tanwar et al., 2010; Izem-Meziane et al., 2012) were included in this meta-analysis.

**FIGURE 6:**
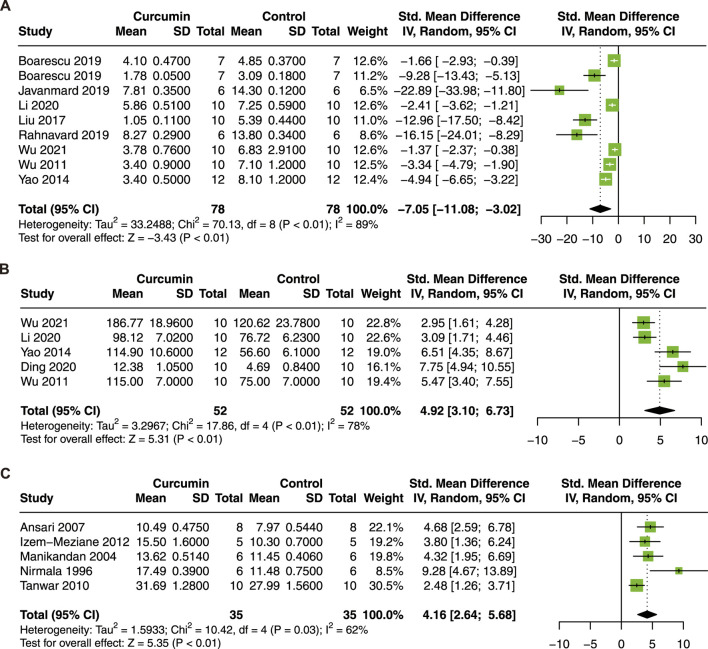
Forest plot showing cardioprotective effects of curcumin on MDA **(A)**, SOD **(B)**, and CAT **(C)** in myocardial ischemia/reperfusion injury animal model. MDA malondialdehyde, SOD superoxide dismutase, CAT catalase.

Curcumin treatment significantly decrease MDA (SMD = −7.05; 95% CI: −11.08, −3.02; *p* < 0.01; I^2^ = 89%; Figure 6A), increase SOD (SMD = 4.92; 95% CI: 3.10, 6.73; *p* < 0.01; I^2^ = 78%; Figure 6B) and CAT (SMD = 4.16, 95% CI: 2.64, 5.69; *p* = 0.03; I^2^ = 62%; Figure 6C) in animal models of myocardial I/R injury.

#### 3.3.5 Inflammation cytokine and apoptosis index

It has been reported that curcumin improves myocardial I/R injury through immunomodulatory effects (Li et al., 2019a). Therefore, we took inflammatory cytokines and myocardial apoptosis index into consideration in this meta-analysis. Four studies with 114 animals for IL-1β (Liu et al., 2018; Boarescu et al., 2019a; Boarescu et al., 2019b; Yan et al., 2021), 4 studies with 110 animals for IL-6 (Wang et al., 2014; Boarescu et al., 2019a; Boarescu et al., 2019b; Yan et al., 2021), and 5 studies with 130 animals for TNF-α (Wang et al., 2014; Liu et al., 2018; Boarescu et al., 2019a; Boarescu et al., 2019b; Yan et al., 2021), and 6 studies with 90 animals for apoptosis index (Izem-Meziane et al., 2012; Yang et al., 2013; Wang et al., 2018b; Liu et al., 2018; Javanmard et al., 2019; Li et al., 2020a) were included in the meta-analysis.

**FIGURE 7:**
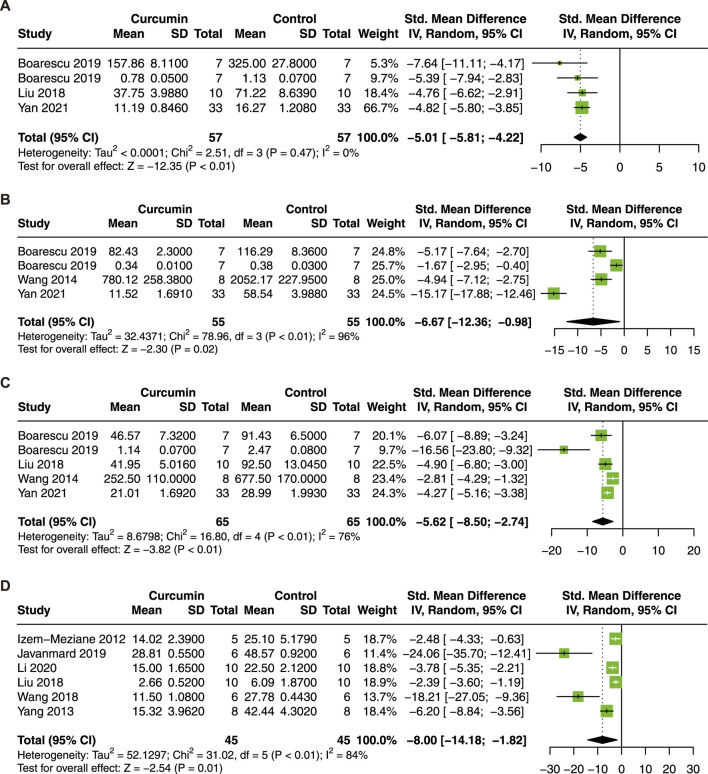
Forest plot showing cardioprotective effects of curcumin on IL-1β **(A)**, IL-6 **(B)**, TNF-α **(C)**, and apoptosis index **(D)** in myocardial ischemia/reperfusion injury animal model. IL-1β, interleukin-1β; IL-6, interleukin-6; TNF-α, tumor necrosis factor-α.

The results demonstrated that curcumin treatment decreased the serum level of IL-1β (SMD = −5.01; 95% CI: −5.81, −4.22; *p* < 0.01; I^2^ = 0%; Figure 7A), IL-6 (SMD = −6.67; 95% CI: −12.36, −0.98; *p* = 0.02; I^2^ = 96%; Figure 7B), TNF-α (SMD = −5.62; 95% CI: −8.50, −2.74; *p* < 0.01; I^2^ = 76%; Figure 7C), and the apoptosis index (SMD = −8.00; 95% CI: −14.18, −1.82; *p* = 0.01; I^2^ = 84%; Figure 7D). Our results suggest that curcumin has significant anti-inflammatory and anti-apoptosis effects in myocardial I/R injury.

## 4 Discussion

### 4.1 Summary of evidence

A total of 38 studies were included in this meta-analysis, and the results suggested that curcumin significantly reduced the myocardial IS, improved cardiac function parameters, downregulated serum myocardial enzyme, improved antioxidant ability, decreased serum inflammatory cytokines, and myocardial apoptosis index. The subgroup analysis of IS revealed that dose of curcumin may significantly influence its therapeutic effect. In the 50–100 mg/kg group, 8 studies using 100 mg/kg curcumin and 1 study using 80 mg/kg curcumin showed favorable therapeutic effects despite high heterogeneity (94%), suggesting that 100 mg/kg may be the most well-documented effective dose for the treatment of myocardial I/R injury.

In conclusion, our results demonstrated a significant cardioprotective effect of curcumin at multiple levels in animal models of myocardial I/R injury. This result provided a solid theoretical basis for subsequent large animal studies and human clinical trials.

### 4.2 Molecular mechanisms

The basic pathological processes of myocardial I/R injury include in inflammation, oxidation stress, cardiomyocyte apoptosis, etc (Schirone et al., 2022). Curcumin has been reported to exert anti-inflammatory, anti-apoptosis, antioxidant, and anti-fibrosis properties (Li et al., 2020b). To better understand the of cardioprotective effect of curcumin in myocardial I/R injury, we summarized its molecular mechanism.

Curcumin reduces myocardial I/R injury through multiple molecular mechanisms, including reducing NF-κB activation (Yeh et al., 2005a; Yeh et al., 2005b), activating the JAK2/STAT3 (Jeong et al., 2012; Liu et al., 2017b) and activating the PI3K/AKT/mTOR signaling pathway (Wu et al., 2021a). In rabbit myocardial I/R injury model, curcumin treatment can significantly inhibit NF-κB activity in myocardial cells, resulting in the downregulation of pro-inflammatory genes such as IL-6, TNF-α, and MCP1, and activation of MMPs (Yeh et al., 2005a; Yeh et al., 2005b). Curcumin activated JAK2/STAT3 pathway, reduced oxidative damage, and inhibited myocardium apoptosis, which improved the myocardial IS in turn (Jeong et al., 2012; Liu et al., 2017b). Further study has implicated pro-surviving pathway of PI3K/Akt/mTOR and MAPK was activated by curcumin, resulting in reduction of myocardial cell apoptosis, and inhibition of inflammation and oxidative stress (Wu et al., 2021a).

Sirtuin 1 (SIRT1), a nicotinamide adenine dinucleotide (NAD)^+^-dependent histone deacetylase, regulates cell stress, energy metabolism, and cell apoptosis through the deacetylation of specific substrate proteins such as NF-κB, FOXO1, TP53, and AMPK, and thus plays a cardiovascular protective role (Yang et al., 2013; Xiao et al., 2016b; Li et al., 2019b; Ren et al., 2020; Aziz et al., 2022). The upregulation and activation of SIRT1 by curcumin pretreatment have been reported to attenuate the mitochondrial oxidative damage, myocardial enzyme levels, and myocardial apoptosis induced by myocardial I/R injury (Yang et al., 2013). Myocardial cells that die after myocardial I/R injury lose their cell membrane integrity, and a measurable infarct area occurs (Liu et al., 2017c). Curcumin maintains mitochondrial membrane stability and reduces mitochondrial oxidative damage by activating SIRT1 (Yang et al., 2013).

Myocardial fibroblasts and macrophages play vital roles in cardiac repair and remodeling after myocardial infarction and affects the prognosis of patients (Prabhu and Frangogiannis, 2016; Wang et al., 2023). Pro-inflammatory cytokines (such as IL-1β and TNF-α) were thought to promote cardiac fibrosis by inducing the expression of TGF-β in fibrogenic macrophages. The expression of IL-18, a pro-inflammatory cytokine, were increased in MI, resulting in upregulation of TGF-β, inflammatory activation, and activation of SMAD2/3 which eventually activated cardiac fibrosis. Curcumin pretreatment inhibited the pro-fibrotic TGFβ1-SMAD2/3 signaling pathway and then improve cardiac fibrosis and cardiac function after MI (Zhao et al., 2022). In addition, TGFβ1-SMAD2/3 signaling can improve cardiac repair after myocardial infarction by inducing myocardial fibroblasts to express CTHRC1 and then selectively activate WNT5A signialing pathway (Wang et al., 2023).

Oxidative stress is involved in the entire process of myocardial I/R injury (Mokhtari-Zaer et al., 2018). Excessive production of reactive oxygen species (ROS) is generally toxic to cells, causing damage to all components of the cell, such as DNA, proteins, and lipids (Bagheri et al., 2016). Therefore, reducing oxidative stress is a strategy to deal with myocardial I/R injury. Curcumin not only protects cardiomyocyte from hypoxia/reoxygenation (H/R) damage by inhibiting the MAPK signaling pathway and Notch pathway to reduce ROS levels (Wei et al., 2019; Zhu et al., 2019), but also alleviates hyperglycemic-induced H9c2 damage by activating Nrf2/HO-1 signaling pathway to reduce ROS production (Wu et al., 2022).

### 4.3 Implications

Animal researches are limited by design variations and methodological flaws. In addition, publication bias also limits the clinical translation of animal studies because positive experimental results are easier to publish (van der Worp et al., 2010). Studies have shown that preclinical animal meta-analysis help the translation of research results from animal findings to clinical trials (Sena et al., 2014). Moreover, the rigor and reproducibility of preclinical animal studies varied greatly. Thus, it is of great importance and necessity to improve the rigor and reproducibility of preclinical research on cardioprotection (Bøtker et al., 2018; Lecour et al., 2021). The consortium for preclinicAl assESsment of cARdioprotective therapies (CAESAR) develops a multicenter, randomized, controlled, clinical trial-like infrastructure to address these problems (Jones et al., 2015). We are looking forward to finding more high-quality preclinical studies under CAESAR.

The results of this study showed that curcumin significantly reduces myocardial IS, improves cardiac function, downregulates myocardial enzyme levels, inhibits oxidative stress, decreased serum inflammatory cytokines, and myocardial apoptosis index to play a cardioprotective role in animal models of myocardial I/R injury. However, the results of this study only used the target animal model and did not include animal models with multiple coexisting diseases, which may differ from the pathology of patients in the actual clinical setting. Although high heterogeneity exists, our results still suggest that curcumin is effective in myocardial I/R injury, and this study can provide a solid theoretical basis for further clinical trials.

### 4.4 Limitations

Firstly, only small animals were used in the included studies. There is a lack of evidence in larger animals. According to the criteria of IMproving Preclinical Assessment of Cardioprotective Therapies (IMPACT) (Lecour et al., 2021), large animal models were the final step of validation to be considered before clinical tests. Therefore, our results might be limited by the lack of large animal models. In addition, most studies used only male animals due to a higher model success rate. Secondly, the heterogeneity in IS, LVDP, CF, CK, CK-MB, and LDH was relatively high. Besides, significant publication bias was found for CK and LDH. Therefore, there is a possibility that the efficacy of curcumin may be overestimated. Finally, all included studies were carried out in target animal models, which were inconsistent with complex cardiovascular comorbidities (such as diabetic hypertension and hyperlipidemia) of patients with myocardial I/R injury in a real-world clinical setting (Andreadou et al., 2021). These factors will greatly limit the extrapolation of our results to clinical translation.

## 5 Conclusion

This meta-analysis suggests that curcumin has excellent potential for the treatment of myocardial I/R injury in animal models. However, this conclusion needs to be further discussed and verified in large animal models and human clinical trials.

## Data Availability

The original contributions presented in the study are included in the article/supplementary material, further inquiries can be directed to the corresponding authors.
